# Impact of Palestine-Israel War on Tunisian People’s Sleep

**DOI:** 10.1192/j.eurpsy.2024.812

**Published:** 2024-08-27

**Authors:** N. Messedi, M. Sehli, F. Guermazi, I. Chaari, F. charfeddine, L. Aribi, J. Aloulou

**Affiliations:** ^1^Psychiatry B, Hedi Chaker University Hospital of Sfax, sfax, Tunisia

## Abstract

**Introduction:**

The Palestine-Israel War has reverberated across borders, transcending boundaries to affect individuals far beyond the conflict zone.

While much attention has been rightfully directed toward the immediate physical and psychological consequences within the war-torn regions, there is a growing need to explore the broader impact on the mental health of populations in neighboring countries including the sleep disorders among the Tunisian population during this war.

**Objectives:**

To study the sleep disorders in Tunisian people related to the extensive war news broadcasting and to identify the factors associated to it.

**Methods:**

It was a cross-sectional, descriptive and analytical study, conducted among Tunisians. Data were collected during October and November 2023, through an anonymous online questionnaire, spread throughout social media (Facebook/Instagram), using the Google Forms® platform. We used a socio-demographic and clinical data sheet and the Insomnia Severity Index (ISI) to measures the severity of insomnia.

**Results:**

A total of 1091 participants completed the questionnaire.The participants’ mean age was 32.7 ± 9.8 years, with a sex ratio (F/M) = 3.5.

The study revealed that 100% of the respondents followed the war, predominantly relying on social media (98.6%) with 55% closely monitoring the war via the media during more than 3 hours per day.

74.1% of the participants were Religious practitioners

According to the (ISI): a significant insomnia was found in 75.2% of participants.

The breakdown of insomnia severity indicated that 47.3% experienced subthreshold insomnia, 25.7% clinical insomnia of moderate severity, and 2.2% clinical insomnia of severe intensity.

The factors significantly associated with severe insomnia were: a male population (p=0.018) and an increase in religious practices (p=0.031).

**Conclusions:**

The impact of the Palestine-Israel war on Tunisian individuals’ sleep patterns, predominantly mediated through increased exposure via social media with using increase in religious practices as a possible coping mechanism.

The study highlights support initiatives to address the psychological repercussions of international conflicts on mental health. This suggests the importance of applying sleep hygiene rules and screening for sleep disorders.

**Disclosure of Interest:**

None Declared

